# Emotional characteristics and intrinsic brain network functional connectivity among adults aged 75+

**DOI:** 10.1093/scan/nsaf017

**Published:** 2025-02-01

**Authors:** Patrick J Pruitt, Kexin Yu, David Lahna, Daniel Schwartz, Scott Peltier, Lisa Silbert, Hiroko Dodge

**Affiliations:** Research Program on Cognition and Neuromodulation Based Interventions, University of Michigan, Ann Arbor, MI 48109, United States; Department of Neurology, Oregon Health and Science University, Portland, OR 97239, United States; Department of Neurology, Oregon Health and Science University, Portland, OR 97239, United States; Department of Neurology, Oregon Health and Science University, Portland, OR 97239, United States; Functional MRI Laboratory, University of Michigan, Ann Arbor, MI 48109, United States; Department of Neurology, Oregon Health and Science University, Portland, OR 97239, United States; Department of Neurology, Massachusetts General Hospital, Harvard Medical School, Boston, MA 02114, United States

**Keywords:** emotional aging, fMRI, brain networks

## Abstract

Despite having a meaningful impact on the quality of life, emotional well-being is often understudied in older adults in favor of cognitive performance, particularly when examining its association with neurobiological function. Socially isolated older adults have poorer emotional health than their non-isolated peers and are at increased risk of dementia. Characterizing neurobiological correlates of emotional characteristics in this population may help elucidate pathways that link social isolation and dementia risk. In a sample of 50 socially isolated older adults aged 75+ years (“older-old”; 30 with mild cognitive impairment; 20 with unimpaired cognition), we use the National Institutes of Health Toolbox—Emotion Battery to examine associations between emotional characteristics and functional magnetic resonance imaging (fMRI)-derived intrinsic brain network functional connectivity. We found a positive association between the default mode network connectivity and negative affect. Amygdala–ventromedial prefrontal cortex (vmPFC) connectivity was negatively associated with psychological well-being and positively associated with negative affect. These results did not survive correction for multiple comparisons. These findings replicate, in a sample of socially isolated older-old adults, the previous work highlighting the relationship between amygdala–vmPFC connectivity and individual differences in emotional health, with more inverse connectivity associated with better emotional characteristics.

## Introduction

Aging is associated with changes to intrinsic brain network functional connectivity (iFC), which may in turn be associated with individual differences in behavioral and psychological characteristics. In the interest of better understanding Alzheimer’s disease and related dementias (ADRDs), much attention has been paid to the relationship between iFC and cognitive performance in older adults. However, the relationship between iFC and emotional characteristics is often overlooked in this population, even though emotional well-being has a comparable impact as cognitive decline on the quality of life.

One challenge in the investigation of emotional well-being is the breadth of tools used to measure emotional constructs, which makes cross-study comparisons difficult. The National Institutes of Health (NIH) have attempted to address this challenge by developing a standardized toolbox of 17 scales measuring different aspects of emotional health, the NIH Toolbox—Emotion Battery (NIHTB-EB; Salsman et al. 2013). From these 17 scales, exploratory and confirmatory factor analyses support three summary scores: negative affect, psychological well-being, and social satisfaction (Babakhanyan et al. 2018). Given that the NIHTB-EB is newly positioned as a “common currency” for measuring emotional well-being, characterizing the brain network correlates of these summary scores in older adults will provide a foundation for investigating the neurobiology of emotional health in aging moving forward. Of particular interest are the so called “older-old” (adults aged 75+ years) with and without cognitive impairment. The investigation of emotional health, brain network function, and cognition in this high-risk population may provide insight into psychosocial factors that contribute to or protect against the development of ADRD and aid in generating prevention approaches.

In understanding the brain network correlates of the emotional characteristics, previous literature primarily implicates three intrinsic networks: the default mode network (DMN), salience network (SN), and cortico–amygdala connectivity. These networks are of interest due to their putative roles in affective and social function. The DMN is thought to facilitate internal mentation (i.e. introspection, mindfulness, and autobiographical memory), which allows for the creation of representations of the self and others (Li et al. 2014). The SN evaluates environmental stimuli for personal relevance, while the amygdala is a canonical structure underlying emotion arousal and processing. Studies of brain function in healthy young adults demonstrated the relevance of these networks to affect: measures of psychological well-being, such as positive affect and general life satisfaction, associate with connectivity measures of the DMN (Luo et al. 2016), SN (Shi et al. 2018, Li et al. 2020), and amygdala (Kong et al. 2015, Sato et al. 2019). As a broad construct negative affect associates with amygdala iFC (Rohr et al. 2015), but to better understand specific aspects of negative affect (e.g. fear, anger, and sadness), it can be informative to look at studies of individuals with affective disorders such as depression and anxiety. These individuals demonstrate altered patterns of DMN (Kaiser et al. 2015), SN (Geng et al. 2015, Hilland et al. 2018), and amygdala (Cullen et al. 2014, Jung et al. 2018, Tang et al. 2018) iFC compared to healthy controls, which can be normalized with treatment (Dodhia et al. 2014, Wang et al. 2016, Hilland et al. 2018). These networks are also implicated in the third NIHTB-EB domain, social satisfaction. Perceived social support associates with DMN (Che et al. 2014) and amygdala (Sato et al. 2019) iFC, loneliness associates with DMN (Spreng et al. 2020) and SN (Layden et al. 2017) iFC, and social rejection recruits regions of the SN (Eisenberger et al. 2003, 2010, Masten et al. 2011) and amygdala (Dewall et al. 2010). Furthermore, the SN is known to be disrupted in disorders of social processing such as autism (Uddin et al. 2013, Margolis et al. 2019). While neuroimaging studies of affective processing have converged upon these three networks, the degree to which network iFC is associated with emotional characteristics in older adults is unclear, despite the vulnerability of this population and potential relevance to cognitive aging.

Although the DMN, SN, and amygdala consistently emerge as correlates of a variety of emotional constructs, other large-scale networks are also reliably found at rest and may be relevant to emotional health in aging. For example, studies examining emotional health have also implicated frontoparietal network (FPN) in emotion regulation (Li et al. 2021) and sensorimotor network (SMN) in emotion recognition (Adolphs et al. 2000, Pitcher et al. 2008, Banissy et al. 2010), although it is unclear to what degree these networks are relevant to the constructs measured by the NIHTB-EB. Therefore, in seeking a broad understanding of how the scales of the NIHTB-EB associate with network iFC in older adults, inclusion of these networks in an exploratory capacity is merited.

Older adults living in social isolation tend to have worse emotional well-being than their non-isolated counterparts and are at higher risks of developing ADRD (Hsiao et al. 2018, Evans et al. 2019, Livingston et al. 2020). In addition to social isolation, perceived isolation, i.e. loneliness is a subjective feeling of the discrepancy between desired social interaction and actual social interaction (Perlman and Peplau 1981). Social isolation and loneliness are highly related but distinct concepts. Loneliness was found to be associated with stronger functional communication in the DMN among middle-aged individuals (Spreng et al. 2020). Loneliness was also related to increased iFC in right central operculum and right supramarginal gyrus among younger adults (Layden et al. 2017). Empirically examining the relationships between emotional well-being and iFC could contribute to understanding the mechanisms underlying the association between social isolation and ADRD. To the authors’ best knowledge, no previous study has investigated the iFC connectivity and NIHTB-EB outcomes among socially isolated older adults, and the current study intends to contribute to the existing literature by studying the relationships using data collected with a sample of older-old adults who meet the operational definition of social isolation.

The primary objective of our investigation is to test associations between NIHTB-EB summary scores and the functional connectivity of intrinsic brain networks most strongly implicated in emotional well-being in previous literature, namely the DMN, SN, and cortico–amygdala connectivity. Our secondary objective is to broadly explore associations between NIHTB-EB scales and iFC of other large-scale intrinsic brain networks including executive control, dorsal attention (DAN), limbic (LIM), somatosensory, and visual (VIS) networks, which may help inform hypotheses of future investigations of emotion–iFC associations. Due to known associations between cognitive functions and iFC (e.g. Damoiseaux et al. 2008, Hausman et al. 2020), we included clinically identified (as opposed to pathologically determined) cognitive status [mild cognitive impairment (MCI) vs. those with normal cognition] as a control variable.

## Methods

### Internet-based Conversational Engagement Clinical Trial

The current study used baseline data from the Internet-based Conversational Engagement Clinical Trial (I-CONECT, ClinicalTrials.gov: NCT02871921). The participants were recruited at two sites [Portland, OR, recruited through the Oregon Health & Science University (OHSU) and Detroit, MI, through the University of Michigan (U-M)] and assessed between July of 2018 and August of 2022. COVID-19 pandemic and the resultant nation-wide social distancing requirement limited our ability to conduct magnetic resonance imaging (MRI). Of 186 participants randomized into the study, 50 participants completed MRI scans before the COVID-19 pandemic social distancing requirement became in-effect and were included in the current study. The I-CONECT trial protocol (Yu et al. 2021) and COVID-19 pandemic-related protocol modifications (Dodge et al. 2024) were documented in detail elsewhere. The study procedures were reviewed and approved by the Institutional Review Board (IRB) at OHSU (IRB STUDY00015937). Individuals were eligible to participate in the I-CONECT study if they were aged 75 or older and socially isolated. The participants were considered socially isolated if they met any one of the following three criteria: (i) scoring <12 on the 6-item Lubben Social Network Scale (LSNS-6) (Lubben et al. 2006), (ii) engaging in conversations lasting 30 min ≦ twice per week, or (iii) answering “often” to at least one question of the three-item UCLA Loneliness Scale (Hughes et al. 2004). Exclusion criteria included having dementia, severe depressive symptoms operationally defined as a 15-item Geriatric Depression Scale score > 7, current alcohol or substance abuse, unstable medical conditions, active systemic cancer within 5 years of the screening visit, or surgery that required full sedation with intubation within 6 months of screening.

### National Institutes of Health Toolbox—Emotion Battery

NIHTB-EB was designed for assessing emotion and behavioral functions of individuals across the lifespan and has been normalized with participants from 3 to 85 years old (Salsman et al. 2013). The NIHTB-EB measures emotion in three domains: psychological well-being, social relationships, and negative affect (*NIH Toolbox Scoring and Interpretation Guide*, n.d.). The domain scores were generated with subscale scores weighted by factor loading and the formula can be found in Babakhanyan et al. (2018). The psychological well-being domain includes subscale measurements of general satisfaction, meaning and purpose, and positive affect. The negative affect domain includes subscales of anger affect, anger hostility, sadness, fear affect, and perceived stress. The social satisfaction domain includes measures on friendship, loneliness, emotional support, instrumental support, and perceived rejection. The perceived rejection subscale was reverse coded so that its direction is consistent with the other subscales within the social satisfaction domain. Each of the domain scores’ general population mean was centered on 50 with a s.d. of 10 (Babakhanyan et al. 2018).

### Control variables

All models controlled for age, sex, site of data collection, motion in MRI scan, social isolation (LSNS-6 < 12), and cognitive status (normal vs. MCI). LSNS-6 defined having a scale score <12 as being socially isolated (Lubben et al. 2006). Although all participants met our operational definition of social isolation, they did not all have a LSNS-6 score < 12. Therefore, we included dichotomously coded LSNS-6 as a control variable. MCI status was determined based on the consensus clinical diagnosis between neurologists and neuropsychologists using the National Alzheimer’s Coordinating Center Uniform Data Set Version 3 (Weintraub et al. 2018, Dodge et al. 2020, Sachs et al. 2020). The participants were blinded of their MCI diagnosis.

### Imaging analysis

Results included in this manuscript come from preprocessing performed using *fMRIPrep* 20.2.6 (Esteban et al. 2019; RRID:SCR_016216), which is based on *Nipype* 1.7.0 (Gorgolewski et al. 2011; RRID:SCR_002502). Preprocessing steps are briefly summarized below; for full details and boilerplate fMRIPrep text, please see Supplementary Methods.

#### Preprocessing

We first estimated susceptibility distortion in the functional BOLD data, using a fieldmap generated from a short reverse phase-encoded BOLD run. We then estimated head-motion with mcflirt (FSL 5.0.9; Jenkinson et al. 2002) and resampled BOLD timeseries in native space with a single, composite transform to correct for both susceptibility distortion and head-motion. BOLD data were then transformed into standard space (*MNI152NLin2009cAsym*) and underwent an ICA-based strategy for Automatic Removal of Motion Artifacts (ICA-AROMA; (Pruim et al. 2015).

#### Regions of interest

Regions of interest (ROIs) for the DMN and SN were defined using the Yeo 7-Network parcellation (Yeo et al. 2011). The amygdala was defined using the atlas in the CONN toolbox (www.nitrc.org/projects/conn, RRID:SCR_009550; Whitfield-Gabrieli and Nieto-Castanon 2012).

#### Individual-level models

We imported preprocessed BOLD and T1w images into CONN toolbox. We removed the first two volumes of each BOLD run to allow for stabilization of signal intensity and applied a 6 mm^3^ Gaussian smoothing filter. Average timeseries for each participant were extracted across all voxels within each ROI. Effects of average gray matter and white matter signal, as well as noise components identified by ICA-AROMA during fMRIPrep preprocessing, were regressed out of the timeseries (i.e. “denoising”). Following denoizing, we proceeded using two different approaches.

For DMN and SN, we calculated ROI-to-ROI connectivity. Pairwise correlations were Fisher-transformed to *Z*-values. Intra-network connectivity strength was calculated as the average pairwise connectivity among regions within a network. This resulted in each participant having two values: one representing their average intra-DMN iFC and another for their average intra-SN iFC.

To investigate cortico–amygdala connectivity, we calculated seed-to-voxel connectivity. Timeseries extracted from the left and right amygdalae were correlated with all other voxels, with results restricted by a cortical mask.

#### Group analyses

To test associations between NIHTB-EB summary scores and iFC in the DMN and SN, we built linear regression models in JASP. In separate models for each network (DMN, SN) and summary scores (positive well-being, social satisfaction, and negative affect; for a total of six regression models), we tested the degree to which the emotional battery summary scores explained network iFC. Bonferroni-adjusted *P*-values for the ROI-to-ROI analyses are corrected for six models.

To test associations between the NIHTB-EB summary scores and cortico–amygdala iFC, we ran separate group-level analyses in CONN toolbox for each summary score, for a total of three voxelwise group-level analyses.

Due to the small sample size, we used a conventional type I error rate of 0.05 to define statistical significance for ROI-to-ROI analyses, and 0.001 for voxelwise analyses. We also included Bonferroni-adjusted *P*-values where relevant so that readers can interpret our results with caution. For voxelwise analyses, Bonferroni correction was applied to clusterwise *P*-values.

For our exploratory analyses, we examined 8 brain networks [7 networks from Yeo et al. 2011 including DMN, SN (labeled ventral attention in Yeo et al.), FPN, DAN, LIM, SMN, and VIS; plus the amygdala seed-to-voxel] and their relationships with 20 behavioral variables from the NIHTB-EB.

## Results

### Sample characteristics

Of the 50 participants whose data were included in the present analyses, 37 were collected at OHSU and 13 at U-M. There were no significant differences in age, sex distribution, or cognitive status distribution between those with MRI assessment (*N* = 50) and those without (*N* = 136); see Supplementary Table 1. Participants with MRI assessment had more years of education (*M* = 15.88 years, s.d. = 2.34) than those without (14.97 ± 2.25; *t*(184) = 2.42, *P* = .017). Seventeen men and 33 women participated; 30 participants were diagnosed with MCI and 20 participants had unimpaired cognition. Participants’ mean age ± s.d. was 80.6  ± 4.4 years. Sex distribution did not differ by site (*χ*^2^(1) = 2.096, *P* = .148), nor did cognitive impairment status (*χ^2^*(1) = 0.934, *P* = .334), age (*t*(48) = 1.288, *P* = .204), or in-scanner motion (mean framewise displacement; *t*(48) = 1.519, *P* = .135). Sex distribution also did not differ by cognitive impairment status (*χ^2^*(1) = 0.238, *P* = .626).

### Hypothesis-testing outcomes

In the ROI-to-ROI analyses, DMN connectivity was associated positively with negative affect (*r*_p_ = 0.357, *P* = .024, *P*_corrected_ = .144).

**Figure 1.**
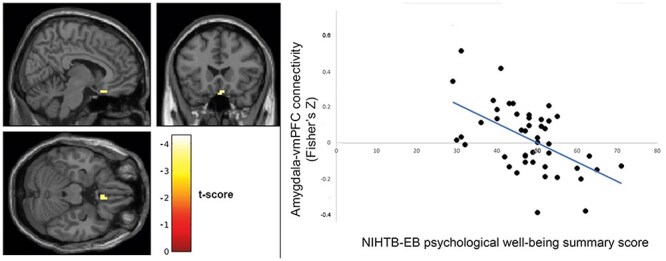
Psychological well-being associates negatively with fronto–amygdala connectivity; left: brain map showing voxels in vmPFC where functional connectivity with amygdala seed correlates significantly with NIHTB-EB psychological well-being summary score (map is thresholded at *P *< .001 uncorrected; colorbar represents *T*-values); right: amygdala–vmPFC connectivity values (extracted from significant cluster at left) correlate negatively with NIHTB-EB psychological well-being summary scores; data shown for descriptive purposes only.

**Figure 2.**
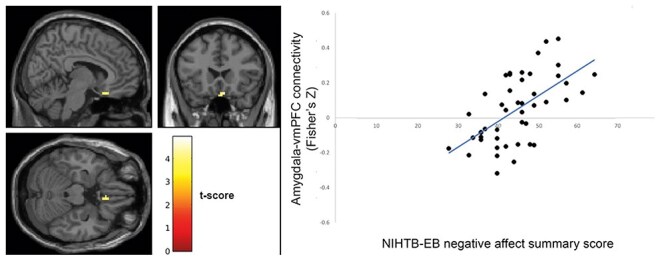
Negative affect associates positively with fronto–amygdala connectivity; left: brain map showing voxels in vmPFC where functional connectivity with amygdala seed correlates significantly with NIHTB-EB negative affect summary score (map is thresholded at *P *< .001 uncorrected; colorbar represents *T*-values); right: amygdala–vmPFC connectivity values (extracted from significant cluster at left) correlate positively with NIHTB-EB negative affect summary scores; data shown for descriptive purposes only.

The seed-to-voxel analysis results showed that psychological well-being was associated negatively with amygdala connectivity with ventromedial prefrontal cortex (vmPFC; Fig. 1; peak *T *= −4.13; Montreal Neurological Institute *xyz*: [−6, 24, −24]; *k *= 24 voxels, *P*_corrected_ = .866). Conversely, negative affect was associated positively with amygdala connectivity with vmPFC (Fig. 2; *T *= 4.26; [4, 22, −22]; *k *= 50, *P*_corrected_ = .255). These results did not survive correction for multiple comparisons.

### Exploratory outcomes

In addition to our hypothesis tests, we ran exploratory analyses to characterize relationships between the summary scales and subscales of the NIHTB-EB and a broad set of intrinsic brain networks. Findings from these analyses are reported in Supplementary Table 2 and Supplementary Fig. 1.

## Discussion

In a sample of older-old adults (75+ years), we measured iFC in several brain networks using resting-state fMRI and emotional characteristics using the NIHTB-EB. We initially examined hypothesized associations between the three NIHTB-EB summary scores, i.e. psychological well-being, negative affect, and social satisfaction, and three brain networks implicated in emotional function, i.e. DMN, SN, and cortico–amygdala connectivity. We found that iFC between the amygdala and a region in the vmPFC is associated negatively with psychological well-being and positively with negative affect. We additionally found a positive association between DMN iFC and negative affect. These findings are in accordance with previous work suggesting a role for the prefrontal cortex in exerting top-down inhibitory control over the amygdala (Motzkin et al. 2015) and the relevance of such control for emotional regulation (Urry et al. 2006), as well as the relevance of the DMN for mood disorders such as depression and subclinical negative affective symptoms (Greicius et al. 2007). Taken together, our work demonstrates that previously identified brain–behavior relationships—namely the associations between DMN/fronto–amygdala networks and emotional function—are present in socially isolated older-old adults and can be found when measuring emotional characteristics using the NIHTB-EB.

In our sample, using a lenient alpha threshold, we found that amygdala–seed connectivity with vmPFC was associated with psychological well-being and negative affect, although neither effect survived multiple comparison correction. Specifically, more negative amygdala–vmPFC functional connectivity was associated with greater psychological well-being and lower negative affect. While functional connectivity analyses are unable to demonstrate the “direction” or “valence” of connectivity (i.e. excitatory or inhibitory), previous investigations into these regions, their anatomical connection, and their role in the neurobiology of emotion regulation, may help illuminate our findings. Previous work has demonstrated the importance of both the amygdala and vmPFC (Nejati et al. 2021) in emotion processing. Notably, these regions are connected structurally through the uncinate fasciculus, forming a frontolimbic circuit in which prefrontal control systems provide inhibitory input to the subcortical LIM system (Mcdonald et al. 1996, Ghashghaei and Barbas 2002). As a result of this inhibitory projection, increased vmPFC activity results in decreased amygdala activity, an inverse coupling which has been demonstrated in neuroimaging task studies of participants engaged in emotion regulation (Urry et al. 2006). Conversely, insufficient activation (or the presence of lesions) in the vmPFC coincides with increased amygdala activity (Motzkin et al. 2015) and may play a role in anxiety disorders such as post-traumatic stress disorder (Etkin and Wager 2007). The inhibitory relationship between vmPFC and amygdala described above would appear as negative functional connectivity between the regions, which we observed in many of the participants in our sample. Our results suggest that effective regulation of the amygdala by the vmPFC, observed as more negative functional connectivity, is associated with better emotional characteristics in our participants (lower negative affect and higher psychological well-being).

Also using a lenient alpha threshold, we additionally found a positive association between DMN connectivity and negative affect, such that higher DMN connectivity was associated with a greater negative affect. Previous work reported a role for DMN hyperconnectivity in negative affect, particularly in the context of mood disorders such as major depressive disorder (MDD). Greater DMN connectivity has been found in participants with MDD relative to controls (Greicius et al. 2007), as well as participants with high familial risk for MDD (Posner et al. 2016), suggesting that this effect is a risk factor that precedes illness onset rather than an effect of the illness. Further, this hyperconnectivity is normalized with effective treatment of MDD by antidepressants (Posner et al. 2013). One potential explanation for these relationships is that DMN hyperconnectivity facilitates (or reflects) maladaptive rumination, in which individuals remain “stuck” engaging in internal, negative self-referential processing and have difficulty transitioning to appropriate, externally focused attention and interaction (Berman et al. 2011, Belleau et al. 2015). In our exploratory analyses, we further found a positive association between DMN connectivity and anger/physical aggression, as well as a negative association between DMN connectivity and the positive affect subscale of the psychological well-being summary score. These results are reported in Supplementary Table 2. This confluence of results further supports the risk of negative affective outcomes associated with DMN hyperconnectivity in older adults.

Notably, we did not find a statistically significant association between SN connectivity and any measures of emotional characteristics. This is surprising, as SN function has previously been associated with measures of psychological well-being, negative affect, and social satisfaction, and found to be altered in affective and social disorders. One potential factor is that relatively little of the previous work linking SN function and emotional health has been done in older adults, and particularly in the older-old. It may be that the role of the SN in some emotional characteristics differs in this population relative to younger adults; further exploration of lifespan differences and changes would be needed to elucidate this possibility. Similarly, 60% of participants in our sample were diagnosed with MCI, and the role of MCI and its frequently comorbid affective impairments (Ismail et al. 2017) on the relationship between SN and emotional characteristics is currently unknown. Unfortunately, our sample is underpowered to examine such an effect, but it remains an intriguing topic for future research. Finally, there is a great deal of heterogeneity in the localization, composition, and naming schemes used for large-scale functional brain networks, including the SN (Uddin et al. 2019). While we used a common parcellation (Yeo 7-Network; Yeo et al. 2011), previous studies examining the relationship between SN function and emotional characteristics have used their own methods of characterizing this network, impacting the size and localization of SN regions, as well as which regions are or are not included in the network. The replicability of network effects across different parcellations is a burgeoning area of interest in the field and identifying when these effects do or not replicate may eventually help us understand more about the networks and behavioral concepts under investigation.

Multiple theories of aging and socioemotional development, such as socioemotional selectivity theory (STT) and selective optimization with compensation (SOC), posit that with the increase of age, individuals often engage in adaptive behaviors to promote successful aging. These behaviors may include choosing to pay attention to more positive emotional experiences and selectively spending more time with close social ties (Carstensen 2006, Baltes and Rudolph 2012). These theories are interesting in the context of the present work in a few ways. First, the connectivity observed between vmPFC and amygdala observed here and in prior work may play a key role in facilitating the selective experience of positive experience, which in turn leads to greater well-being and lower negative affect. Second, it is interesting to consider these theories in the context of social isolation. Older adults tend to experience more positive emotions than their younger adult counterparts, perhaps as a result of the previously mentioned adaptive behaviors. However, when faced with long-term unavoidable stress, such as widowhood or caregiving, age-related advantages in emotion processing can be compromised or even reversed (Charles et al. 2009). Social isolation can also be a chronic stressor in older adulthood, and chronic stress can similarly facilitate or exacerbate social isolation. Whether social isolation reflects a failure of adaptive behaviors posited by STT and SOC, or a successful narrowing of social contacts to only those that are most beneficial, may differ across individuals. Further consideration of these ideas will be aided by future work examining these brain–behavior relationships in older adults both with and without social isolation.

The association between friendship and amygdala–dorsomedial PFC connectivity is a finding of exploratory analysis. We are cautious about overinterpretation and intentionally did not emphasize it as a “found” relationship. We hope this preliminary finding with our unique sample of socially isolated older adults can inform future hypothesis-driven investigations. The results need to be replicated in future studies. Therefore, we believe it is not yet appropriate to integrate this exploratory finding with theories of aging and socioemotional development.

In addition to our hypothesis-driven analyses, we also took an exploratory approach to investigating associations between iFC in a broad set of networks and both the summary and subscales of the NIHTB-EB. While we did not approach these tests with specific hypotheses, our hope was that they may assist in future confirmatory research.

The study contributes to the existing literature by empirically examining the association between NIHTB-EB outcomes and selected brain network connectivity among a socially isolated older-old study sample. Socially isolated older adults, as a “hidden” population that is difficult to recruit, have been not only under-researched with neuroimaging methodology but also at high risk of cognitive decline and onset of dementia. A better understanding of the emotion function and brain connectivity could help identify psychobehavioral pathways that could explain variations of brain functioning in later life. NIHTB-EB was designed to be a comprehensive tool for easy comparison between age groups, allowing future studies to take a life course perspective and compare the current study findings with individuals of different life stages and various backgrounds. Despite these strengths, the study findings need to be interpreted with caution due to a few limitations. First, this study uses single-timepoint data; therefore, the study findings are correlational in nature and cannot be used to infer causal relationships between network iFC and emotional characteristics. Second, the current study uses resting-state, or “task-free,” fMRI to characterize brain network function. However, some aspects of brain function relevant to emotional characteristics may emerge only when probed using emotion-eliciting tasks in-scanner. Further research with different modalities, including task fMRI, therefore could be helpful for examining the robustness of our results. Third, the sample size of the study is small, especially when investigating brain–behavior relationships. Given the small sample size, we adopted more lenient alpha thresholds without adjusting for multiple comparisons for the confirmatory type of analyses, which increases the risk of making type I error. Underpowered samples may also inflate effect sizes of brain–behavior relationships (Marek et al. 2022). Despite this, we would note that the current findings regarding relationships between negative emotion and amygdala/vmPFC connectivity, as well as DMN connectivity, are in accordance with prior literature and thus provide converging evidence for such effects. The sample size limited our ability to stratify our sample by cognitive impairment, leaving this as a potential confound in our analyses. However, the socially isolated older adults who make up our sample are, by definition, a difficult population from which to recruit, and we hope these results provide a valuable, if limited, insight into the neural correlates of their affective characteristics. Further investigation, with the above limitations in mind, is needed to support or refute the study results, and they should be interpreted with appropriate caution in the meantime.

These results characterize associations between brain network connectivity at rest and emotional characteristics, in a sample drawn from a unique and important population and using a recently established NIHTB of emotional measurements. We hope they serve as a foundation or reference point for future brain–behavior association studies using the NIHTB-EB.

## Supplementary Material

nsaf017_Supp

## Data Availability

Data will be shared upon request.
